# Chronic almond nut snacking alleviates perceived muscle soreness following downhill running but does not improve indices of cardiometabolic health in mildly overweight, middle-aged, adults

**DOI:** 10.3389/fnut.2023.1298868

**Published:** 2024-01-08

**Authors:** Leah Siegel, Jessica Rooney, Lindsey Marjoram, Lauren Mason, Elena Bowles, Thomas Valente van Keulen, Carina Helander, Vernon Rayo, Mee Young Hong, Changqi Liu, Shirin Hooshmand, Mark Kern, Oliver C. Witard

**Affiliations:** ^1^Centre for Human & Applied Physiological Sciences, Faculty of Life Sciences and Medicine, King’s College London, London, United Kingdom; ^2^School of Exercise and Nutritional Sciences, San Diego State University, San Diego, CA, United States

**Keywords:** functional foods, muscle damage, exercise tolerance, body composition, appetite

## Abstract

**Introduction:**

As a popular food snack rich in protein, fiber, unsaturated fatty acids, antioxidants and phytonutrients, almond nut consumption is widely associated with improvements in cardiometabolic health. However, limited data exists regarding the role of almond consumption in improving exercise recovery. Accordingly, we aimed to investigate the impact of chronic almond snacking on muscle damage and cardiometabolic health outcomes during acute eccentric exercise recovery in mildly overweight, middle-aged, adults

**Methods:**

Using a randomized cross-over design, 25 mildly overweight (BMI: 25.8 ± 3.6 kg/m^2^), middle-aged (35.1 ± 4.7 y) males (*n* = 11) and females (*n* = 14) performed a 30-min downhill treadmill run after 8-weeks of consuming either 57 g/day of whole almonds (ALMOND) or an isocaloric amount (86 g/day) of unsalted pretzels (CONTROL). Muscle soreness (visual analogue scale), muscle function (vertical jump and maximal isokinetic torque) and blood markers of muscle damage (creatine kinase (CK) concentration) and inflammation (c-reactive protein concentration) were measured pre and post (24, 48, and 72 h) exercise. Blood biomarkers of cardiometabolic health (total cholesterol, triglycerides, HDL cholesterol, and LDL cholesterol), body composition and psycho-social assessments of mood (POMS-2 inventory), appetite and well-being were measured pre and post intervention.

**Results:**

Downhill running successfully elicited muscle damage, as evidenced by a significant increase in plasma CK concentration, increased perception of muscle soreness, and impaired vertical jump performance (all *p* < 0.05) during acute recovery. No effect of trial order was observed for any outcome measurement. However, expressed as AUC over the cumulative 72 h recovery period, muscle soreness measured during a physical task (vertical jump) was reduced by ~24% in ALMOND vs. CONTROL (*p* < 0.05) and translated to an improved maintenance of vertical jump performance (*p* < 0.05). However, ALMOND did not ameliorate the CK response to exercise or isokinetic torque during leg extension and leg flexion (*p* > 0.05). No pre-post intervention changes in assessments of cardiometabolic health, body composition, mood state or appetite were observed in ALMOND or CONTROL (all *p* > 0.05).

**Conclusion:**

Chronic almond supplementation alleviates task-specific perceived feelings of muscle soreness during acute recovery from muscle damaging exercise, resulting in the better maintenance of muscle functional capacity. These data suggest that almonds represent a functional food snack to improve exercise tolerance in mildly overweight, middle-aged adults.

## 1 Introduction

The manifestation of exercise-induced muscle damage (EIMD) principally results from the active lengthening of skeletal muscle fibers during eccentric muscle contractions (1), and is commonly experienced by novice exercisers unaccustomed to muscle loading exercise. While muscle strain, local inflammation and the production of reactive oxygen species all play a role in facilitating muscle adaptation to exercise training (2), minimizing excessive muscle soreness during the initial 48–72 h following exercise serves as a crucial psycho-physiological determinant of adherence to regular physical activity in recreational exercisers (3). Intuitively, by alleviating the perception of muscle soreness, an individual’s tolerance to participate in subsequent bouts of activity is improved, thus promoting a more physically active lifestyle. Hence, from a behavioral perspective, interventions to combat EIMD are fundamental to maintaining physical activity levels across the general population.

Multiple studies in healthy trained and untrained populations have examined the efficacy of various nutritional strategies to facilitate muscle recovery following muscle damaging exercise, reporting mixed findings (4). In terms of athletic populations, the International Olympic Committee has recognized vitamin D, omega-3 polyunsaturated fatty acids, and anti-inflammatory supplements such as curcumin as evidence-based, potentially effective, nutritional interventions to promote exercise recovery and enhance training capacity (5). More recently, and of greater practical relevance to the general public, studies have taken a food first approach by examining the efficacy of functional foods to promote exercise recovery. For instance, bovine milk (6, 7) and tart cherry juice (8, 9) ingestion was shown to confer beneficial outcomes in terms of reducing muscle soreness, the better maintenance of muscle function, and ameliorating the rise in putative blood markers of muscle damage (i.e., creatine kinase, CK) inflammation and oxidative stress following muscle damaging exercise. These data have provided the impetus for investigating the efficacy of alternative functional foods to promote exercise recovery across multiple populations.

Whole nuts such as almonds represent an increasingly popular choice of food snack and are often included in healthy eating guidelines. In practice with regards to dietary snacking behaviors, there is a concerted effort to displace more commonly consumed unhealthy snack foods that are low in fiber and rich in saturated fatty acids, refined starch and added sugar, particularly in overweight and obese populations. In contrast, almonds are rich in protein, fiber, unsaturated fatty acids, multiple micronutrients (vitamin E, magnesium, riboflavin, copper, niacin, and manganese), and a host of phytonutrients, and contain only trace amounts of saturated fatty acids (10). This nutrient profile has been shown to confer clinical benefits for almond consumption over more commonly consumed snack foods (11), without leading to an increase in body weight. Improvements in cardiometabolic risk factors include an increased endothelial function, reduced circulating low-density lipoprotein cholesterol (LDL-C) (12, 13), improved glycemic control (14) and an increase in heart rate variability during mental stress (15).

Almond consumption also has been demonstrated to promote lower food consumption (16) and greater faecal energy losses (17, 18). Accordingly, recent research reported that the daily incorporation of 15% of energy from almonds as part of a 12-week weight loss diet enhanced the loss of total and trunk adipose tissue in compliant individuals (19). Moreover, a 6-week intervention of 57 g of almonds per day yielded reduced abdominal and leg fat (12) and waist circumference (12, 20) in overweight individuals. Thus, it has been speculated that these effects occur through control over food cravings (21), as mediated via enhanced blood glucose regulation.

Scientific rationale also exists linking almond consumption with exercise tolerance, specifically with regards to acute recovery from muscle damaging exercise. As a protein dense plant food, almonds provide ~6 g of protein per 30 g (1 oz) serving, including an abundant supply of the branched-chain amino acids that have been reported to ameliorate muscle soreness following eccentric exercise (22–24). Moreover, the antioxidant content of almonds has been implicated in reducing oxidative damage (25) and the accompanying inflammatory response (26), thus resulting in the alleviation of delayed onset of muscle soreness (27). Accordingly, a recent study by Nieman et al. (28) reported that 4 weeks of daily (57 g) almond ingestion ameliorated the increase in serum CK concentration as a putative and indirect biomarker of muscle damage during the initial 24 h post eccentric exercise in mildly overweight, middle-aged men and women. However, no changes in muscle soreness or muscle function were observed in this study, likely owing to the relatively short (4 week) intervention period.

Therefore, the aims of this study are two-fold. First, to investigate the impact of 8 weeks of whole almond snacking on indices of muscle damage during acute recovery from eccentric-based downhill running exercise. Second, to investigate the impact of 8 weeks of almond snacking on changes in body composition and cardiometabolic health markers. A participant cohort of mildly overweight, middle-aged adults was recruited to maximize the muscle damaging effect of eccentric exercise. We hypothesized that 8 weeks of snacking with 57 g/day of whole almonds would improve indices of cardiometabolic health and promote exercise recovery, as determined by an alleviation of muscle soreness, attenuated rise in blood markers of muscle damage and inflammation, and maintenance of muscle functional capacity in comparison to an isocaloric (86 g/day) control condition of pretzel snacks.

## 2 Materials and methods

### 2.1 Participants and study design

Thirty participants were recruited to participate in this study which received local research ethics committee approval (ID: 21319). Unfortunately, 5 participants withdrew from the trial citing issues unrelated to the study design leaving a final cohort of n = 25 (11 male and 14 female) middle-aged (35.1 ± 4.7 y) mildly overweight (BMI: 25.8 ± 3.6 kg/m_2_) adults (Table 1). A power calculation (G*Power version 3.1) conducted *a priori* based on He et al. (27) suggested that a sample size of 20 participants (effect size: 2.6; power 0.80) would be sufficient to detect a difference in muscle soreness and plasma CK concentration between conditions. Given the extensive range of secondary outcome measurements included in the present study, and our commitment to conducting a robust study, we recruited a total of 25 participants. Eligible participants were not using any nutritional supplements that could impact antioxidant or inflammatory status within a month preceding the trial. Other exclusion criteria included musculoskeletal limitations, use of anti-inflammatory medications and smoking.

**Table 1:** Participant characteristics and habitual dietary intake (*n* = 25, 11 males and 14 females).

Characteristics	Value
Age (years)	35.1 ± 4.7
Stature (cm)	171.7 ± 9.6
Body mass (kg)	68.0 ± 13.4
BMI (kg/m_2_)	25.8 ± 3.6
VO_2peak_ (mL/kg/min)	48.3 ± 9.4
Daily step count	11,358 ± 4,922
Dietary carbohydrate (g/day)	230 ± 45
Dietary protein (g/day)	45 ± 14
Dietary fat (g/day)	75 ± 10

Values are expressed as means ± SD.

**Figure 1:**
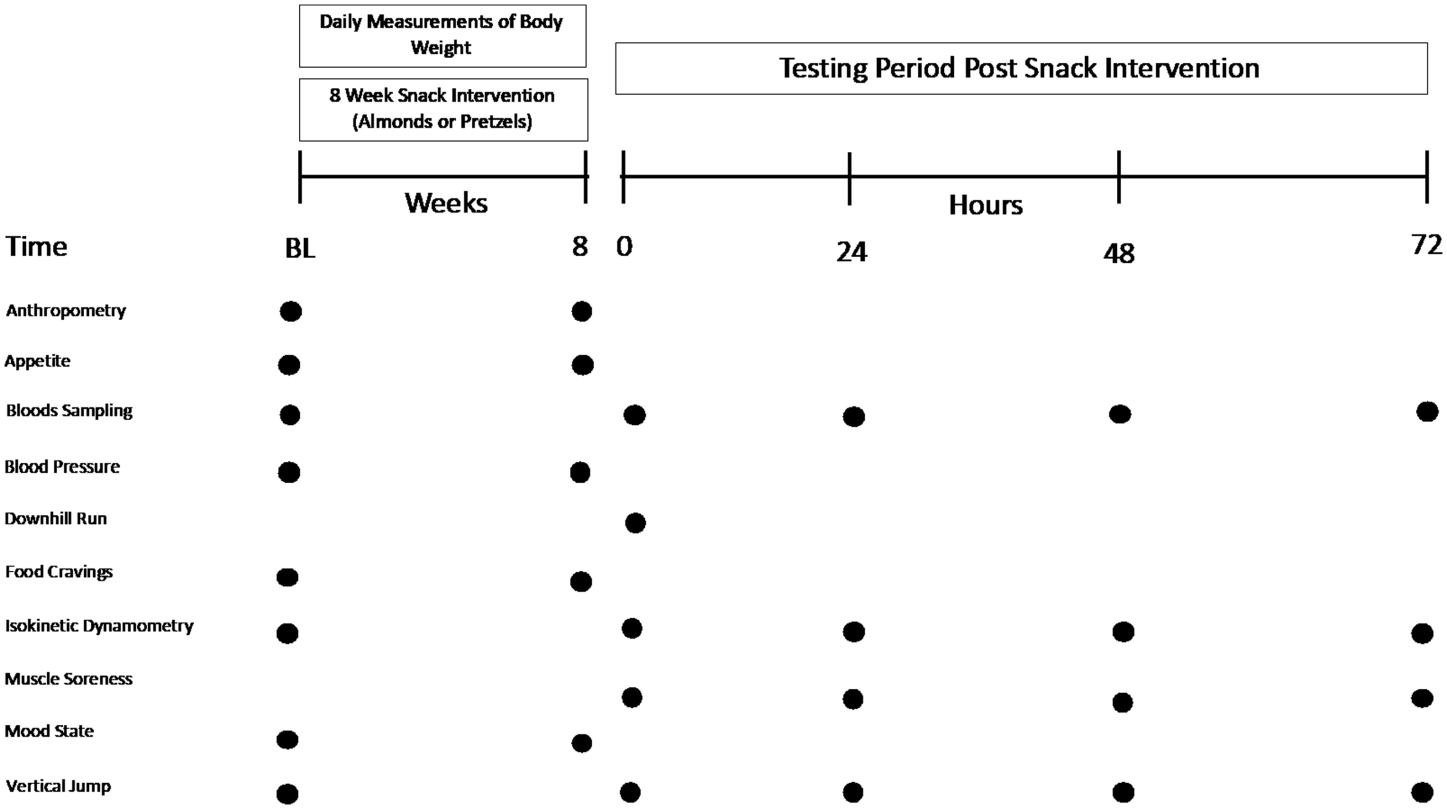
Overview of study protocol.

The study utilized a randomized, crossover research design with two experimental arms. Each trial consisted of a 30-min downhill treadmill run to induce muscle damage which was conducted after 8 weeks of consuming 57 g/day of whole (shelled, unskinned and raw) almonds (ALMOND) or an isocaloric quantity (86 g/day) of pretzels (CONTROL, Table 2). Trials were separated by at least 3 weeks. Muscle soreness was assessed on a range of lower limb muscle groups, muscle maximal torque production was evaluated for knee extensors and flexors at two contraction velocities (60°/sec and 120°/sec), and measurements of vertical jump performance, blood indices of muscle damage (creatine kinase concentration) and blood indices of inflammation (c-reactive protein concentration) were recorded prior to downhill treadmill running (baseline) and at 24-h, 48-h and 72-h post exercise. The order of conducting all outcome measurements was standardized for all participants at all timepoints, as listed below. Pre and post 8 weeks of supplementation, blood samples also were analyzed for glucose, insulin and lipid concentrations and measurements of body composition and blood pressure were obtained (Figure 1).

**Table 2:** Nutrient composition of one serving of raw almonds (57 g) and a calorie matched serving of pretzels (86 g).

Nutritional variable	57 g raw almonds	86 g pretzels
Energy (kcal)	324	327
Protein (g)	11.9	9.0
Total fat (g)	28.0	2.2
Saturated fat (g)	1.9	0.4
Carbohydrate (g)	12.1	69.0
Fiber (g)	7.0	2.6
Sugars (g)	2.4	2.4
Calcium (mg)	80.1	69.0
Iron (mg)	1.4	0.3
Sodium (mg)	1	1,089
Potassium (mg)	430	117

### 2.2 Study foods

Raw, shelled whole almonds (unsalted) were generously provided by the Almond Board of California. Almonds were conveniently portioned into 57 g servings and individually packed prior to shipment by ABC. Unsalted pretzels (Snyders®) were purchased and packed by investigators in a resealable, snack-sized paper bag. A lab-grade scale (Ohaus Corporation, Pine Brook, United States) was used to weigh out pretzels. No stipulations were made regarding the timing of snack intake during the day. Participants were instructed to refrain from consuming additional nuts and seeds over the 8-wk period.

### 2.3 Preliminary assessments

At least 1-wk prior to the 8-wk supplementation period, participants were familiarized with all outcome measurements and the muscle damage protocol (see details below). Anthropometric measurements of body mass and stature were collected using a set of scales (SECA, UK) and wall mounted stadiometer, respectively prior to conducting the familiarization downhill run.

Participants undertook a VO_2peak_ test to determine the relative exercise intensity of the muscle damage protocol. Following a 5-min self-selected warm up, participants began running at 7 km/h for 1-min followed by an increase of 1 km/h increments at 1 min intervals until reaching a speed of 15 km/h. Once a 1 min period had been completed at 15 km/h, treadmill (Kettler run77) speed remained constant and the treadmill gradient was elevated by 1% each minute until voluntary exhaustion. We recorded continuous breath-by-breath measurements of VO_2_ throughout the exercise duration via a metabolic analyzer (Quark CPET, Cosmed, Rome, Italy). HR was recorded continuously using a radiotelemetry HR monitor (POLAR® chest strap) RPE was recorded throughout the test using the Borg scale. VO_2_ values of the last 15 s of each stage were averaged. Once the participant reached voluntary exhaustion (signaled by a raised hand), the protocol was stopped and a VO_2peak_ was established.

Habitual dietary intake was assessed over 3 days during the preliminary period using Nutritics software, and this analysis revealed that participant’s diets contained a total dietary carbohydrate of 230 ± 45 g/day, dietary protein of 45 ± 14 g/day, and dietary fat of 75 ± 10 g/day. Participants replicated dietary intake prior to trials and when questioned did not report any major deviations in dietary macronutrient content or total calorie intake (Table 1).

### 2.4 Downhill running protocol

The muscle damage protocol consisted of a 30-min downhill treadmill run, as described previously (29, 30). Participants maintained a steady-state HR during the downhill run at a − 10% gradient. Target HR during the downhill run was set at predicted 70% VO_2peak_, as calculated via regression analysis by plotting the individualized HR-VO_2_ relationship determined during the preliminary VO_2peak_ test. Accordingly, treadmill speed was adjusted to maintain a constant HR throughout the two trials. Heart rate was monitored to ensure the pre-determined target exercise intensity was achieved. Participants were prohibited from alcohol and caffeine intake and abstained from exercise over the 48 h period prior to the downhill run. RPE was measured using the modified Borg scale at 5-min intervals during exercise (31) and water was consumed *ad libitum*.

### 2.5 Pre, peri and post supplementation measurements

Body mass, body composition [bioelectrical impedance (TANITA Body Analyzer) and waist circumference] and blood pressure were measured in the fasted state pre and post supplementation in both trials. Measurements of physical activity (using actigraph), mood state (POMS-2 inventory), appetite and food cravings [by questionnaire (21)] were conducted throughout the 8-week. supplementation period. Participants were instructed to maintain their habitual diet over the 8-week snacking period and completed food frequency questionnaires (FFQs) at the start and end of the snacking period to monitor compliance.

### 2.6 Biochemical indices of cardiometabolic health, muscle damage and inflammation

Blood samples were drawn in the overnight fasted state from a forearm vein pre and post 8 weeks of almond (ALMOND) or pretzel (control) consumption and dispensed into potassium EDTA and plain serum vacutainer tubes. EDTA tubes were kept on ice and then spun (3,000 rpm) at 4°C and plasma was stored at −80°C until analysis. Serum/plasma concentrations of insulin, glucose, triglycerides, total cholesterol, high-density lipoprotein cholesterol (HDL-C) and LDL-C were measured pre and post intervention. Insulin concentrations were converted from μIU/mL to pmol/L using a correction factor of 1 μIU/mL = 6 pmol/L (32). Insulin sensitivity was calculated using the QUICKI method. Blood samples collected immediately prior to downhill running (0 h) and at 24, 48, and 72 h post-exercise timepoints were analyzed for serum c-reactive protein and plasma creatine kinase and concentrations. All assays were performed using commercially available colorimetric or immunometric kits.

### 2.7 Muscle soreness

Perceived muscle soreness of the quadriceps, hamstring, gluteal, gastrocnemius and tibialis anterior muscle groups was measured using a validated visual analogue scale (VAS) (33). Briefly, participants marked their soreness rating on a 100 mm line between two anchor points that represented no pain (far left) or most pain ever experienced (far right). Muscle soreness was measured with participants in the following positions: the knee joint flexed at 90° (knee flexion), extended to 0° (knee extension) and general soreness of specific muscle group without manipulation (quadriceps, hamstring, gluteus maximus, gastrocnemius and tibialis anterior). Muscle soreness ratings were also collected during the conduct of a vertical jump and assessment of peak isometric torque on the isokinetic dynamometer.

### 2.8 Muscle function

Peak isokinetic torque was measured using an isokinetic dynamometer (KinKom) at two contraction speeds (60 and 120°/sec) for knee extension and knee flexion of dominant and non-dominant legs. Following a 10-min standardised stretching-based warm up, three maximal contractions were separated by a 30 s rest interval and assessed at each contraction speed for each leg, with the best effort recorded. Vertical jump performance (best of 3 attempts) was assessed prior to the exercise bout and at each post-exercise time point using a Takei Jump Meter with the participants’ hands located on hips during a countermovement jump (Takei Scientific Instruments Co., Ltd., Tokyo, Japan). A 30-s rest period was standardized between repetitions.

### 2.9 Data presentation and statistical analysis

All time-dependent data were analyzed using two factor (time and trial) repeated measures ANOVA. Initial analysis examined for order effects in the model. Where no order effect was observed this variable was removed from the model and main effects of trial and time and their interactions were examined. The time factor includes two levels (Pre and Post) for anthropometric, psychological and psycho-social measurements and four levels for measurements of blood indices of muscle damage, systemic inflammation, muscle soreness and muscle function. The trial factor included two levels (ALMOND and CONTROL) for all measurements. In the event of significant interactions, a Bonferroni *post hoc* test was used to detect at what timepoint differences lie. All time-independent data (HR, RPE and variables expressed as tAUC) were analysed using Student’s paired t-test (2 tailed). An advantage of using tAUC is that both the magnitude of response (i.e., overall increase in plasma creatine concentration following exercise compared with baseline) and the changes over time (i.e., time intervals between consecutive measurement of CK concentration) are accurately captured, irrespective of nonuniform time intervals between measurements. Statistical significance was set at an ἀ-level of *p* < 0.05.

## 3 Results

Participant characteristics are presented in Table 1. No order effect was observed for any outcome measurement across trials.

### 3.1 Anthropometric, physiological, psychological and psychosocial assessments

No main effects of time, trial or time × trial interactions were observed for body weight, BMI, waist circumference, percentage body fat (total and trunk), and systolic blood pressure over the 8-week intervention period (all *p* > 0.05). A main effect (decrease) of time was observed for body fat % (arms) and body fat % (legs, both *p* < 0.05), but no significant trial or time × trial interaction was detected (*p* > 0.05, Table 3).

**Table 3:** Pre-post intervention changes in anthropometric measurements over 8-week intervention period.

Muscle group	Trial	Pre	Post	*p* value
Body weight (kg)	Almond	68.3 ± 14.0	68.7 ± 13.5	
	Control	68.0 ± 13.4	68.7 ± 13.7	0.652
BMI (kg/m_2_)	Almond	23.1 ± 3.7	23.2 ± 3.6	
	Control	23.0 ± 3.6	23.5 ± 3.9	0.352
Waist circumference (cm)	Almond	78.3 ± 9.1	78.4 ± 9.0	
	Control	78.3 ± 8.8	78.4 ± 8.9	0.689
Body fat % (total)	Almond	23.2 ± 8.4	21.7 ± 6.9	
	Control	24.6 ± 7.9	22.1 ± 6.4	0.577
Body fat % (arms)	Almond	21.4 ± 10.7	19.7 ± 9.2	
	Control	20.9 ± 10.7	19.0 ± 8.8	0.534
Body fat % (legs)	Almond	25.6 ± 11.1	23.9 ± 10.3	
	Control	27.7 ± 10.6	23.9 ± 10.7	0.439
Body fat % (trunk)	Almond	21.6 ± 7.1	21.0 ± 6.3	
	Control	22.0 ± 7.7	21.1 ± 7.7	0.523
LBM (kg)	Almond	50.0 ± 9.9	51.2 ± 9.9	
	Control	49.4 ± 10.5	51.7 ± 10.8	0.296
BP (systolic)	Almond	119.3 ± 10.6	117.4 ± 11.9	
	Control	120.0 ± 11.4	117.9 ± 12.7	0.990
BP (diastolic)	Almond	79.8 ± 10.8	78.2 ± 9.5	
	Control	81.1 ± 10.4	78.1 ± 9.2	0.564

Values are expressed as means ± SD. *p* values refer to time × trial interactions.

No differences in average HR (CONTROL 132 ± 12 bpm, ALMOND: 130 ± 12 bpm, *p* > 0.05) or RPE (CONTROL: 17 ± 2, ALMOND: 18 ± 3, *p* > 0.05) were observed between trials over the 30-min downhill treadmill run. No main effects of time, trial, or time × trial interactions were observed for any *psychological* assessment of mood state over the 8-week intervention period (all *p* > 0.05, Table 4). No main effects of time, trial, or time × trial interactions were observed for any *psycho-social* assessment of appetite, well-being, or food cravings over the 8-week intervention period (all *p* > 0.05, Tables 5, 6).

**Table 4:** Pre-post intervention changes in *psychological* measurements of mood state over the 8-week intervention period.

Cluster (T score)	Trial	Pre	Post	P value
TMD	Almond	51.7 ± 9.9	50.9 ± 9.1	
	Control	51.7 ± 9.7	50.8 ± 6.7	0.635
Anger-Hostility	Almond	46.5 ± 7.7	46.4 ± 8.3	
	Control	46.5 ± 7.0	45.7 ± 6.6	0.263
Confusion-Bewilderment	Almond	51.3 ± 7.0	49.5 ± 8.1	
	Control	51.5 ± 9.0	49.3 ± 5.7	0.235
Depression-Dejection	Almond	47.4 ± 6.3	47.4 ± 7.0	
	Control	47.8 ± 7.4	47.4 ± 5.5	0.456
Fatigue-Inertia	Almond	47.0 ± 9.2	45.5 ± 7.7	
	Control	46.5 ± 8.1	45.2 ± 5.3	0.198
Tension-Anxiety	Almond	47.0 ± 9.2	45.5 ± 7.7	
	Control	46.5 ± 5.3	45.2 ± 5.3	0.325
Vigor-Activity	Almond	47.5 ± 10.2	48.4 ± 9.0	
	Control	48.2 ± 8.3	46.6 ± 8.1	0.135
Friendliness	Almond	48.9 ± 9.5	48.8 ± 8.4	
	Control	46.9 ± 9.8	47.1 ± 10.1	0.325

TMD, total mood disturbance. Values are expressed as means ± SD. *p* values refer to time × trial interactions.

**Table 5:** Pre-post intervention changes in *psycho-social* assessments of appetite and well-being over the 8-week intervention period.

Domain	Trial	Pre	Post	*p* value
Hunger	Almond	48 ± 4	49 ± 3	
	Control	50 ± 4	50 ± 4	0.637
Fullness	Almond	51 ± 4	55 ± 4	
	Control	51 ± 3	56 ± 3	0.562
Happiness	Almond	67 ± 18	67 ± 12	
	Control	62 ± 16	65 ± 13	0.745
Anxiety	Almond	42 ± 24	40 ± 17	
	Control	46 ± 19	42 ± 18	0.642
Alertness	Almond	59 ± 19	59 ± 16	
	Control	54 ± 14	58 ± 16	0.511
Contented	Almond	64 ± 17	64 ± 15	
	Control	60 ± 16	64 ± 17	0.856

Values are expressed as means ± SD. *p* values refer to time × trial interactions.

**Table 6:** Pre-post intervention changes in *psycho-social* assessments of appetite and well-being over the 8-week intervention period.

Domain	Trial	Pre	Post	*p* value
Desire for sweet foods	Almond	42 ± 24	44 ± 17	
	Control	45 ± 24	43 ± 22	0.523
Desire for savory foods	Almond	52 ± 18	49 ± 16	
	Control	58 ± 15	51 ± 24	0.747
Frequency of food cravings	Almond	47 ± 20	48 ± 16	
	Control	51 ± 22	50 ± 18	0.236
Strength of food cravings	Almond	47 ± 21	44 ± 17	
	Control	49 ± 20	53 ± 19	0.524
Difficulty in resisting food cravings	Almond	46 ± 21	41 ± 19	
	Control	47 ± 21	47 ± 19	0.329
Eating frequency due to cravings	Almond	51 ± 20	46 ± 19	
	Control	52 ± 20	48 ± 20	0.083
Frequency of cravings for chocolate	Almond	41 ± 22	36 ± 19	
	Control	41 ± 26	39 ± 19	0.441
Frequency of cravings for other sweets	Almond	38 ± 21	43 ± 19	
	Control	40 ± 21	41 ± 21	0.254
Frequency of cravings for fruit	Almond	44 ± 21	38 ± 19	
	Control	37 ± 23	40 ± 20	0.269
Frequency of cravings for dairy	Almond	38 ± 24	32 ± 20	
	Control	37 ± 26	36 ± 23	0.499
Frequency of cravings for starchy foods	Almond	56 ± 18	49 ± 19	
	Control	51 ± 17	49 ± 20	0.195
Frequency of cravings for savory foods	Almond	43 ± 19	41 ± 18	
	Control	54 ± 19	48 ± 19	0.352
Difficulty in controlling eating	Almond	35 ± 22	34 ± 17	
	Control	37 ± 21	37 ± 18	0.618

Values are expressed as means ± SD. *p* values refer to time × trial interactions.

### 3.2 Cardiometabolic health markers

Whereas no main effects of time or trial were observed for LDL concentration, a significant time × trial interaction (*p* < 0.05) was detected. However, Bonferroni *post hoc* was unable to detect any statistically significant pre-post changes in LDL for either ALMOND OR CONTROL. No main effects of time, trial, or time × trial interactions were observed for any other cardiometabolic health marker over the 8-week intervention period (all *p* > 0.05, Table 7).

**Table 7:** Pre-post intervention changes in blood markers of cardiometabolic health over the 8-week intervention period.

Metabolite	Trial	Pre	Post	*p* value
Glucose (mg/dL)	Almond	90.0 ± 8.7	89.8 ± 10.8	
	Control	92.6 ± 8.9	87.4 ± 1.9	0.325
Insulin (pmol/L)	Almond	34.2 ± 25.2	39.0 ± 39.9	
	Control	34.2 ± 27.6	27.0 ± 26.4	0.524
Insulin sensitivity	Almond	0.39 ± 0.05	0.39 ± 0.06	
	Control	0.39 ± 0.06	0.44 ± 0.12	0.253
Total cholesterol (mg/dL)	Almond	162.1 ± 24.5	156.5 ± 27.7	
	Control	159.0 ± 28.2	158.6 ± 28.6	0.451
HDL-C (mg/dL)	Almond	56.6 ± 11.5	58.7 ± 12.0	
	Control	56.9 ± 12.7	56.1 ± 11.0	0.651
TG (mg/dL)	Almond	88.4 ± 47.1	87.2 ± 40.5	
	Control	95.5 ± 47.2	80.2 ± 25.0	0.129
LDL-C (mg/dL)	Almond	87.8 ± 29.8	80.4 ± 27.8	
	Control	82.9 ± 30.3	86.5 ± 29.6	0.050

Values are expressed as means ± SD. *p* values refer to time × trial interactions.

### 3.3 Muscle damage and inflammation

**Figure 2:**
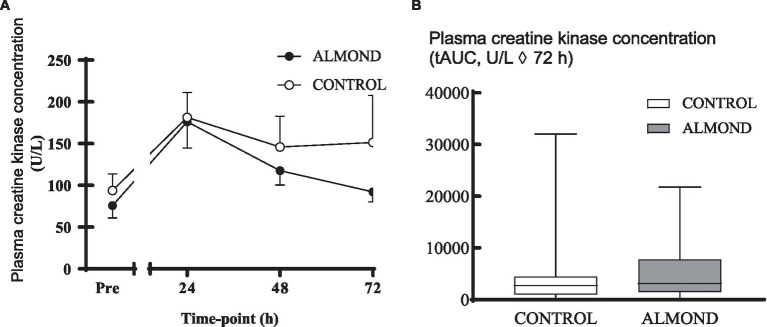
Plasma creatine kinase concentration at baseline (pre) and during the 72-h recovery period following downhill running **(A)** and over the 72 h period expressed as tAUC **(B)**. Data are analysed by two factor repeated measures ANOVA with time and treatment as within-subject factors **(A)** or by Student’s paired t-test (2 tailed). Data are expressed as means ± SEM.

**Figure 3:**
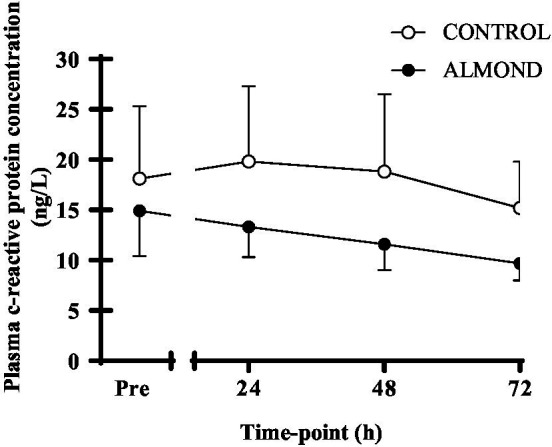
Plasma c-reactive protein concentration at baseline (pre) and during the 72-h recovery period following downhill running. Data are analyzed by two factor repeated measures ANOVA with time and treatment as within-subject factors. Data are expressed as means ± SEM.

A significant time effect was observed for plasma CK concentration (*p* < 0.05), but no main effects of trial or time **×** trial interactions were observed (*p* > 0.05). Moreover, no difference in tAUC for the cumulative 72 h CK response was observed between trials (*p* > 0.05, Figure 2). No main effects of time, trial or time **×** trial interactions were observed for plasma CRP concentration when expressed as raw data over time (*p* > 0.05, Figure 3) or total antioxidant capacity (*p* > 0.05, data not shown).

### 3.4 Muscle soreness

**Figure 4:**
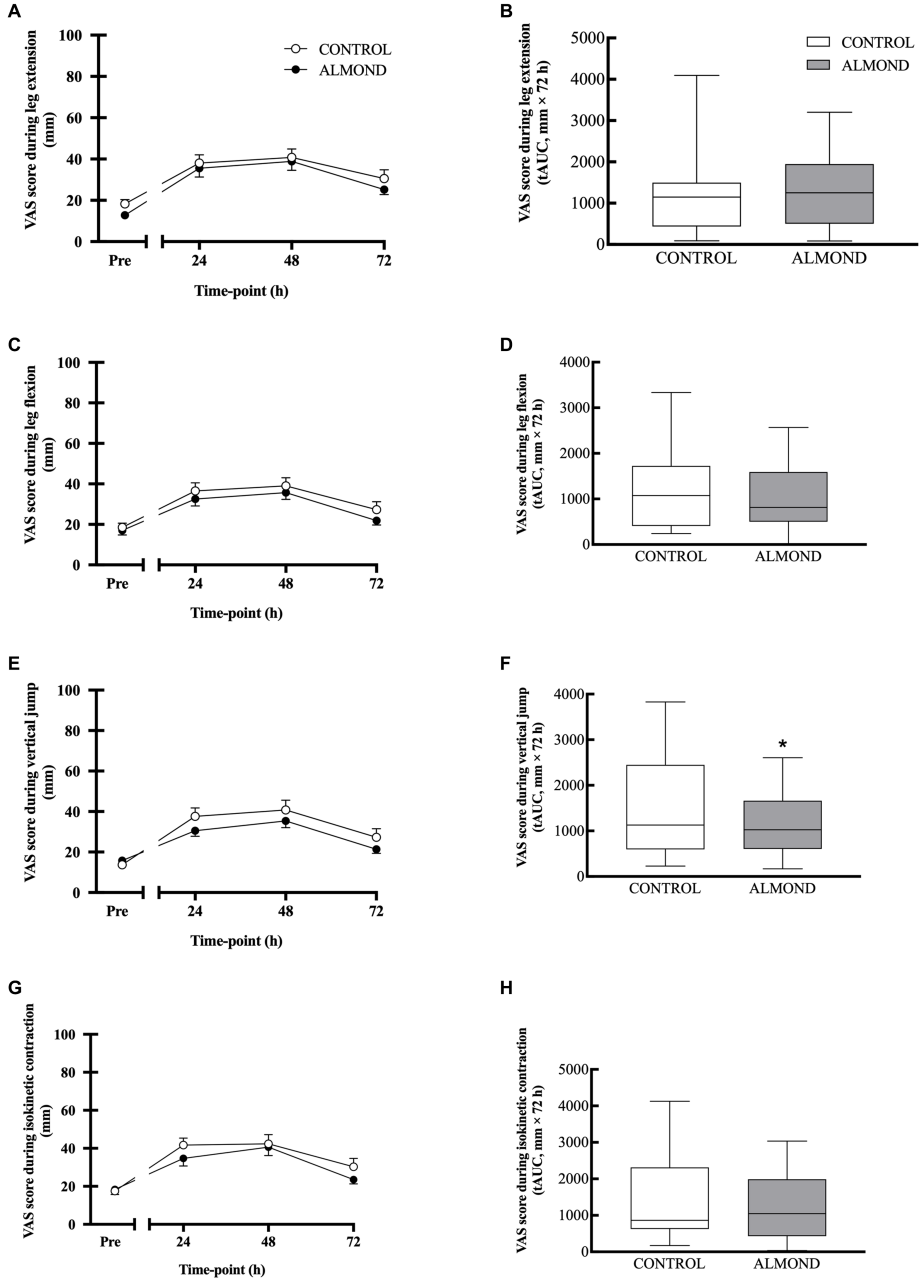
General muscle soreness (0–100 scale) with leg in extension **(A,B)** and flexion **(C,D)** positions, or during vertical jump **(E,F)** and isokinetic dynamometry **(G,H)** during the 72-h recovery period following downhill running. Data are analyzed by two factor repeated measures ANOVA with time and treatment as within-subject factors **(A,C,E,G)** or by Student’s paired t-test (2 tailed) **(B,D,F,H)**. Data are expressed as tAUC over the cumulative 72 h recovery period with box and whisker plots. * denotes significant difference from CONTROL (*p* < 0.05). Data are expressed as means ± SEM.

Statistical analysis of subjective measurements of muscle soreness, expressed as raw data values across time, detected a significant time effect (*p* < 0.05) across all domains (i.e., soreness during leg extension, soreness during leg flexion), but no main effects of trial or time **×** trial interactions were observed for any domain (*p* > 0.05, Figure 4). However, expressed as tAUC for the cumulative 72 h recovery period, muscle soreness when conducting a vertical jump activity was reduced by 24% in ALMOND vs. CONTROL (*p* < 0.05, Figure 4F). Expressed as tAUC, soreness scores for the left gastrocnemius and tibialis anterior muscle groups were not different between ALMOND vs. CONTROL trials (*p* = 0.09).

### 3.5 Muscle function

**Figure 5:**
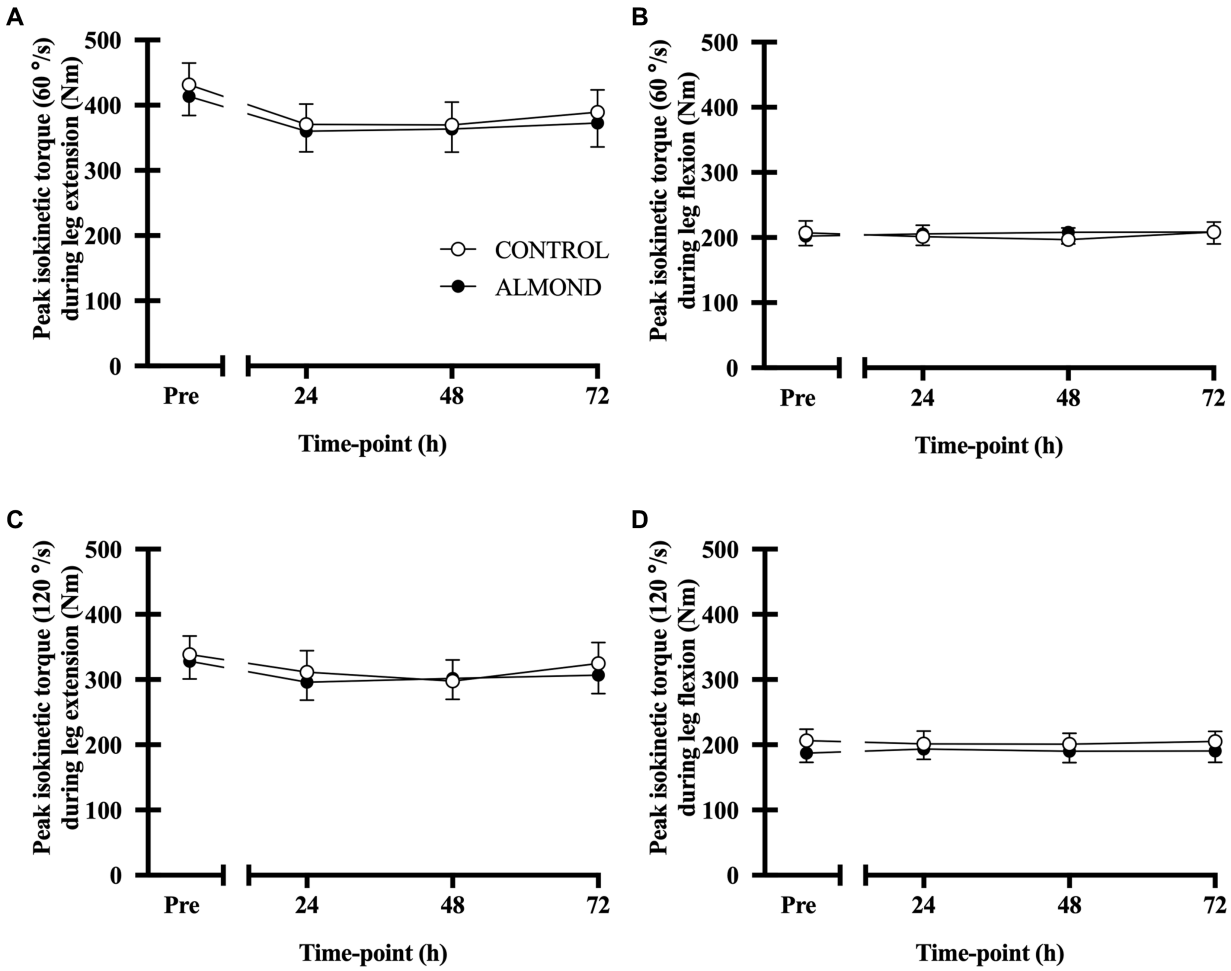
Peak isokinetic torque during leg extension **(A,C)** and leg flexion **(B,D)** at baseline (Pre) and during the 72-h recovery period following downhill running. Measurements conducted at 60°/sec and 90°/sec contraction speeds. Data are analyzed by two factor repeated measures ANOVA with time and treatment as within-subject factors. Data are expressed as means ± SEM.

**Figure 6:**
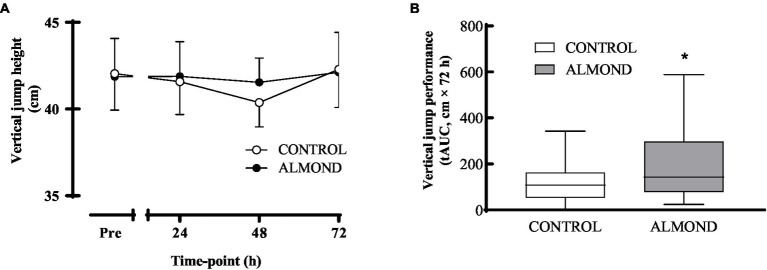
Vertical jump height during the 72-h recovery period following downhill running. Data are analyzed by two factor repeated measures ANOVA with time and treatment as within-subject factors **(A)** or by Student’s paired t-test (2 tailed) **(B)**. Data are expressed over time **(A)** or as tAUC over the cumulative 72 h recovery period with box and whisker plots **(B)**. *Denotes significant difference from CONTROL (*p* < 0.05). Data are expressed as means ± SEM.

No main effect of time, trial or time × trial interactions were observed for peak (Figure 5) or average (data not shown) isokinetic torque during leg extension and leg flexion (all *p* > 0.05). Expressed as data values across time, a significant time effect (*p* < 0.05) was detected for vertical jump height, but no main effects of trial or time **×** trial interactions were observed (*p* > 0.05). However, expressed as tAUC over the cumulative recovery period, vertical jump performance was better maintained in ALMOND vs. CONTROL (*p* < 0.05, Figure 6).

## 4 Discussion

This study investigated the influence of chronic almond consumption on body composition, cardiometabolic health markers, psycho-social assessments of mood, appetite and well-being, and indices of muscle damage following intense eccentric-based exercise in mildly overweight, middle-aged adults. The 30-min downhill running protocol was effective, albeit to a moderate degree, in eliciting a temporary state of muscle damage, as evidenced by a transient rise in plasma creatine kinase concentration, an increased perception of muscle soreness and impairment in vertical jump performance during the acute 72 h recovery period. Whereas no difference in creatine kinase response to exercise was observed between experimental (ALMOND) and control (CONTROL) conditions, the perception of muscle soreness during a physical task (vertical jump) was reduced following 8 weeks of almond consumption and muscle function was better maintained at the 48 h time point of exercise recovery. However, no impact of chronic almond consumption was observed on assessments of body composition, appetite, cardiometabolic health, mood or well-being in this cohort of mildly overweight, middle-aged, men and women. Taken together, these data provide preliminary experiential evidence to support the notion that almonds may serve as an effective functional food snack to facilitate recovery from muscle damaging exercise, thus potentially improving compliance to exercise programmes in untrained individuals. Assuming these findings can be replicated within a laboratory setting and translated into the field, a logical follow up study includes the refinement of daily dose and duration of almond snacks for improving outcomes related to exercise recovery across various populations.

The observation that 8 weeks of almond snacking resulted in an amelioration of perceived feelings of muscle soreness following eccentric-based downhill running exercise is consistent with previous studies that administered pistachio nuts as snacks in physically-active young men (34). However, we contend that the mediators of candidate anti-inflammatory mechanisms of action likely differ between these two functional foods, at least in terms of reducing muscle soreness. In the present study, based on the measurement of serum c-reactive protein concentration as an independent biomarker of inflammation, we reported no effect of almond consumption on the inflammatory response to exercise. Unfortunately, the measurement of other inflammatory markers (i.e., cytokines) was beyond the scope of our experiment. Nonetheless, recent evidence suggests a role for oxylipins in modulating the inflammatory response to exercise (35, 36), and specifically DiHOMES that are widely recognized to confer beneficial biological outcomes. In this regard, almond ingestion has been shown to modulate the oxylipin response to muscle damaging exercise. For instance, Nieman et al. (28) demonstrated that 4 weeks of almond consumption increased plasma levels of 12,13-DiHOME and decreased plasma levels of 9,10-DiHOME in response to an intense bout of resistance-based, multi-component, eccentric exercise in untrained middle-aged adults (28). These data suggest that 12,13-DiHOME serves as a lipokine that is elevated in response to exercise and exhibits favorable biological actions on metabolic health, energy regulation (37–39) and muscle soreness during exercise recovery. Conversely, pistachio supplementation was associated with a marked increase in plasma 9,10-DiHOME concentration with negligible effect on 12,13-DiHOME concentration following prolonged exercise (40). This observation was related to high levels of raffinose in pistachios and their translation from the colon to the circulation during exercise. Given that almonds exhibit low levels of raffinose, the decline in 9,10-DiHOME levels was not unexpected in the study by Nieman et al. (28). Taken together, these data indicate that different anti-inflammatory mechanisms likely underpin the protective effect of almond and pistachio nut snacking on exercise-induced muscle soreness. Future mechanistic studies are warranted to fully elucidate the potential role of almonds in the context of exercise recovery across multiple physically active populations.

The effect of almond snacking in reducing perceived feelings of muscle soreness during a physical task (vertical jump) translated, at least partially, to improved muscle function during acute exercise recovery in our cohort of mildly overweight, middle-aged, adults. In this regard, the decline in vertical jump performance following muscle damaging exercise was mitigated with almond supplementation at the 48 h timepoint post downhill running. This functional effect of almond consumption on muscle functional capacity is particularly noteworthy given that only a moderate degree of muscle damage, as evidenced by a relatively modest increase in plasma creatine kinase concentration, was induced by the downhill running protocol in this participant group. Interestingly, our previous observation of a reduced muscle soreness response to muscle damaging exercise with pistachio ingestion was not accompanied by the better maintenance of vertical jump performance during exercise recovery. The most likely reason for these discrepant findings relates to participant characteristics given that physically active individuals recruited in our previous studies (34, 41) were likely more accustomed to running exercise than the current cohort, and thus recorded lower perceived soreness ratings and negligible impairments in muscle functional capacity following muscle damaging exercise. In contrast, no changes in muscle strength measured by isokinetic dynamometry were observed during exercise recovery in either ALMOND or CONTROL. Given this observation, we speculate that improvements in isokinetic torque would have been more likely in the setting of a more severe muscle damage protocol, i.e., drop jumps or isokinetic dynamometry. Moreover, this null effect of almond snacking on muscle function (based on peak and average isokinetic torque), combined with a reduced soreness response following almond ingestion, provides further evidence of an inflammatory response to muscle damaging exercise, despite the negligible change in serum c-reactive protein response. Theoretically, a blunted inflammatory response to tissue disruption/damage may serve to reduce nociceptor stimulation (42) and, by extension, reduced perceptions of muscle soreness. Although there was no effect of almond supplementation on the selected blood marker of inflammation in the present study, further studies are warranted to explore the influence of almond consumption on other inflammatory mediators such as leukotrienes, eicosanoids, prostaglandins, and thromboxanes. This potential anti-inflammatory action of almond ingestion may be of particular interest to novice exercisers who undertake intense or unaccustomed bouts of exercise.

A secondary aim of the present study was to investigate the impact of chronic almond snacking on cardiometabolic health outcomes, alongside body composition and psycho-social assessments of mood, appetite and well-being. Overall, we failed to detect any pre-post almond ingestion changes in blood lipid profiles (total cholesterol, triglycerides, HDL-C and LDL-C) and insulin sensitivity vs. control, and all measured parameters of body composition remained constant over the 8-week supplementation period. Moreover, no changes in mood state, appetite or well-being were detected over the intervention period based on qualitative analyses of validated questionnaires. A key factor in detecting an intervention effect on secondary outcome variables relates to the characteristics of participants recruited in the investigation. We aimed to recruit overweight, middle-aged participants who were not involved in any structured exercise training programme. In the present study, our participant sample may be deemed in the early stages of middle-age (35.1 ± 4.7 years), only mildly overweight with a BMI of >25 kg/m_2_, and physically active albeit untrained. Moreover, baseline levels of total cholesterol, triglycerides, HDL-C and LDL-C were within a normal healthy range. Hence, evaluating the effects of almond ingestion in a fundamentally healthy population likely limited our capacity to detect statistically significant, physiologically-relevant changes in cardiometabolic health outcomes. According to a recent meta-analysis, nut consumption (including almonds) promotes beneficial effects on metabolic biomarkers (i.e., reduced total cholesterol levels) in obese participants that were metabolically impaired at baseline (43). Hence future long-term studies are warranted into the efficacy of almond supplementation to improve cardiometabolic health outcomes in populations that exhibit a greater cardiometabolic risk profile or suffer from metabolic disease (11).

A primary strength of the present study was novelty in terms of investigating a bonafide functional food that could be prescribed relatively simply as a snack into the habitual diet, rather than isolated supplement. Moreover, we implemented downhill running as an ecologically valid mode of eccentric-based exercise rather than previously employed models of muscle damage that include isokinetic dynamometry (23, 44), multi-component resistance exercise (28), and box jumps (45). One limitation of the study was the randomized cross-over trial design. The key issue with muscle damage protocols designed in a cross-over fashion concerns the potential of experiencing a repeated bout effect (46). In this regard, participants may adapt to a single bout of eccentric exercise and therefore gain some protection against muscle damage in subsequent eccentric exercise bouts. However, this repeated bout effect is likely to be more relevant in the context of severe muscle damage situations such as eccentric exercise using isokinetic dynamometry. Moreover, the more modest damage induced by downhill running may mitigate the impact of a repeated bout effect. To explore the potential for a repeated bout effect impacting our primary endpoints (i.e., blood markers of muscle damage, muscle soreness and muscle function), we tested for an order effect in the trial responses for all endpoints and observed no significant order effects for these key outcomes in our analysis. Hence, we have confidence that the order in which participants undertook the trials did not influence the results. This observation indicates that any significant outcomes observed are not due to the order of trials, but more likely by the almond intervention itself. Finally, given the relatively long-term nature of the nutritional intervention (i.e., 8 weeks), it was not feasible to control background diet and instead habitual diet was monitored using food frequency questionnaires.

## 5 Conclusion

Eight weeks of daily (57 g/day) almond snacking may provide some alleviation of muscle soreness and the better maintenance of explosive power (as determined by vertical jump performance) during acute recovery from muscle damaging exercise in mildly overweight, middle-aged, adults. Hence, in terms of practical implications, our data provide preliminary experiential evidence that almond snacking may help promote adherence to novel training programmes in population groups that are not necessarily accustomed to exercise training. Additional studies are warranted to elucidate the mechanism(s) that underpin this apparent beneficial effect of almond snacking on exercise recovery in more compromised overweight and obese populations.

## Data availability statement

The raw data supporting the conclusions of this article will be made available by the authors, without undue reservation.

## Ethics statement

The studies involving humans were approved by King’s College London Research Ethics Committee. The studies were conducted in accordance with the local legislation and institutional requirements. The participants provided their written informed consent to participate in this study.

## Author contributions

LS: Data curation, Investigation, Writing – review & editing. JR: Data curation, Formal analysis, Project administration, Software, Writing – review & editing. LiM: Investigation, Writing – review & editing. LaM: Investigation, Writing – review & editing. EB: Investigation, Writing – review & editing. TK: Investigation, Writing – review & editing. CH: Investigation, Writing – review & editing. VR: Investigation, Writing – review & editing. MH: Investigation, Writing – review & editing. CL: Investigation, Writing – review & editing. SH: Investigation, Writing – review & editing. MK: Conceptualization, Funding acquisition, Methodology, Project administration, Writing – review & editing. OW: Conceptualization, Data curation, Funding acquisition, Project administration, Supervision, Writing – original draft, Writing – review & editing.
